# Synergistic Effects of Micro-electrolysis-Photocatalysis on Water Treatment and Fish Performance in Saline Recirculating Aquaculture System

**DOI:** 10.1038/srep45066

**Published:** 2017-03-27

**Authors:** Zhangying Ye, Shuo Wang, Weishan Gao, Haijun Li, Luowei Pei, Mingwei Shen, Songming Zhu

**Affiliations:** 1 College of Biosystems Engineering and Food Science, Zhejiang University, Hangzhou 310058, China; 2 College of Engineering, Anhui Agricultural University, Hefei 230036, China

## Abstract

A new physico-chemical process for TAN (total ammonia nitrogen) removal and disinfection is introduced in saline recirculating aquaculture system (RAS), in which the biofilter is replaced with an integrated electrolysis cell and an activated carbon filter. The electrolysis cell which is based on micro current electrolysis combined with UV-light was self-designed. After the fundamental research, a small pilot scale RAS was operated for 30 days to verify the technical feasibility. The system was stocked by 42 GIFT tilapia (*Oreochromis niloticus*) fish with the rearing density of 13 kg/m^3^. During the experiments, the TAN concentration remained below 1.0 mg/L. The nitrite concentration was lower than 0.2 mg/L and the nitrate concentration had increased continuously to 12.79 mg/L at the end. Furthermore, the concentration of residual chlorine in culture ponds remained below 0.3 mg/L, ORP maintained slight fluctuations in the range of 190~240 mV, and pH of the water showed the downtrend. Tilapia weight increased constantly to 339.3 ± 10 g. For disinfection, the active chlorine generated by electrochemical treatment caused *Escherichia coli* inactivation. Enzyme activity assay indicated that the activity of glutamate dehydrogenase, carbonic anhydrase and glutamic pyruvic transaminase increased within the normal range. The preliminary feasibility was verified by using this physico-chemical technology in the RAS.

In recirculating aquaculture system(RAS), the metabolites, food remnants, biological body and other proteins of cultured species can not be decomposed in time, leading to a rising ammonia, nitrite nitrogen and other harmful substances in water bodies, which will pollute the water environment and produce toxic effects on cultured species, causing a large number of animal death^1^,^2^,^3^. In this regard, during the process of industrial aquaculture, the traditional way of water treatment is the application of biological purification technology. The bio-filter which is the core part of RAS is widely used. Biological treatments are carried out by bacteria that are attached to biological carrier, which convert TAN and nitrate to nitrogen gas so as to achieve water purification^4^. Despite their widespread application, both nitrification and denitrification suffer from certain shortcomings.

The following disadvantages are generally associated to biological processes in connection with RAS operation: (1) requiring long startup procedures and a long recovery period following failure; (2) autotrophic bacteria characterized by long doubling times and low bacterial yield; (3) high sensitivity to fluctuations in temperature and water alkalinity; (4) entailing very large reactors to oxidize typical RAS ammonia fluxes^5^,^6^,^7^. Concurrently, biological treatments also have other drawbacks. Therefore, it is high time to adopt some new technology to solve these problems^8^.

With the continuous development of electrode manufacturing technology, electrochemical oxidation which is regarded as advanced oxidation technology has received widespread concern on wastewater treatment in recent years. This approach plays a significant role in wastewater treatment, which devotes to degrade ammonia, nitrite nitrogen, organic matter etc.^9^,^10^,^11^. There are many applications of electrochemical oxidation technology in wastewater treatment over recent years, such as the treatment of difficultly degradable organic industry wastewater^12^, ammonia of hoggery sewerage^13^, municipal sewage^14^, printing and dyeing wastewater^15^, landfill leachate^16^, tannery wastewater^17^. The method that takes advantage of high anode potential and catalytic activity makes organic pollutants directly oxidized in water, or produces strong oxidants such as hydroxyl radical to degrade the organic matter in water.

In a direct anodic oxidation process, ammonia is directly absorbed on the anode surface and lose electrons to be oxidized. The overall electrochemical reaction of ammonia to nitrogen is generally considered to be a three-electron exchange reaction (Equation (1))^18^,^19^,^20^:


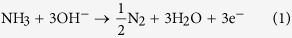


There are two essential conditions for the direct electrochemical oxidation of ammonia. Firstly, alkaline media will be needed to ensure that ammonia is mostly in the presence of free NH_3_; the other is a suitable anode potential.

In an indirect oxidation process, strong oxidants are generated through anode reaction, which can realize degradation and removal of ammonia. For example, in the chloride-containing wastewater, the electro-generated active chlorine is produced in the bulk of solution via electrochemical reactions of chloride ion. The active chlorine, including chlorine, hypochlorite ion and hypochlorous acid, is the main oxidant for ammonia removal^21^,^22^. At this point, the mechanism of indirect ammonia oxidation process is described as the stoichiometric reactions (Equations (2, 3, 4, 5, 6))^23^, which is similar to the “breakpoint chlorination” mechanism.

Dominant reaction on the anode:


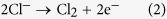


Dominant reaction in the aqueous phase:


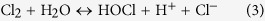



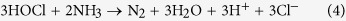



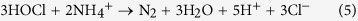



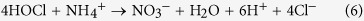


In addition, chlorination and UV disinfection are two of the most common applications in drinking water and wastewater. In many UV disinfection installations, free chlorine is present in the water as it passes through UV reactors^24^. A major advantage of this combined process is that the UV driven chlorine process, as an advanced oxidation process, can be a treatment option for disinfection by-products (DBPs) which are produced during chlorine disinfection, and can be used to inactivate water-borne pathogenic microorganisms and to destroy hazardous organic compounds in drinking water and wastewater^25^. It has been reported that hypochlorous acid can dissociate to hydroxyl radical (·OH) andchlorine radical (·Cl) upon absorption of UV photon in the 200–400 nm regions (Equation (7))^26^,^27^:


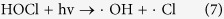


Hydroxyl will induce a series of radical chain reaction and attack the organic pollutants in water. Its ultimate production is CO_2_, H_2_O and few inorganic salts, which realize zero pollution and zero discharge of waste. It also has been pointed out that Cl radicals may play an important role for the chemical transformation of organic substances in marine environments^28^. Therefore, it is promising to treat water and wastewater with the aid of these radicals. Nevertheless, there are little researches about the applications of electrolysis combined with UV-light on water treatment in the treatment of low concentrations of ammonia in saline RAS.

This study addresses the effect of electrochemical oxidation technology combined with low-power and low-pressure mercury lamp for treating low concentration of ammonia in aquaculture water, so as to explore cooperative technology between micro electrolysis and UV-light for practical aquaculture water treatment. By researching the variation of water quality parameters and fish performance between simulated wastewater and practical aquaculture water during the water treatment process of synergies in RAS, it has been proved that there are technical feasibilities which combine micro current electrolysis with UV-light on water treatment in saline RAS. It is hoped that the cooperative technology can supersede the traditional biological filter and apply to marine RAS so as to promote sustainable development of fishery.

## Results and Discussion

### Variation of water quality parameters in simulated seawater during fundamental research

**Figure 1:**
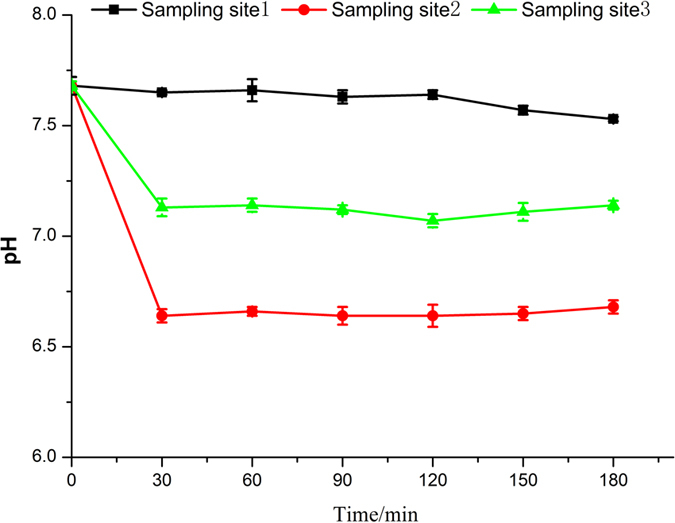
Change in pH of simulated seawater during electrolysis process.

As shown in Fig. 1, pH rapidly decreased to about 6.64 when the simulated aquaculture water flowed through the electrolysis cell. In this process, active chlorine was produced on the electrolyzer’s anode via electrochemical oxidation. When the effluent of electrolysis cell flowed into buffer pool, due to the mixture of two parts (the effluent of electrolysis cell and water flow directly coming from breeding tanks) pH remained at around 7.15. Then water in buffer pool flowed into the activated carbon filter. Perhaps due to the adsorption of residual chlorine and the impurities (such as trace metal oxides) existing in artificial salt which caused a reduction in hydrogen ions, the pH value increased to a certain degree. But it will be slightly lower than the initial pH value before electrolysis. On the whole, during the 3 h of electrolysis action, pH decreased from 7.68 to 7.53 in the breeding tanks. During electrolysis process, the active chlorine, including chlorine, hypochlorite ion and hypochlorous acid, would react with ammonia rapidly. At pH ≥ 7, HOCl (and/or OCl^−^) which is the major oxidizing species in the classical chloramination process (Equation (5)). Due to the low pH and high chloride ions concentration in the anode vicinity, the primary oxidizing agent is Cl_2_ which reacts with ammonia in the near anode area (Equation (8))^23^.


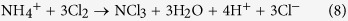


According to Eqs. (5), (8), we could conclude that pH value decreased more obviously with the extension of electrolysis time during the electro-oxidation process of ammonia. Excessive acidity of the seawater not only will damage the fish body tissue, but also can make the pH value decrease in fish blood and weaken its ability to carry oxygen, resulting in hypoxia^29^. Therefore, it is necessary that baking soda or medical stone should be added into the system to adjust the pH of aquaculture water and ensure the stability of pH during the small pilot experiment^29^,^30^.

**Figure 2:**
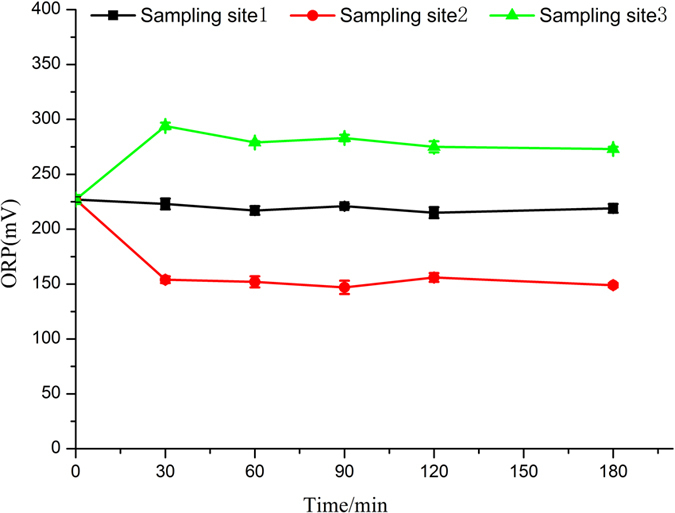
Change in ORP of simulated seawater during electrolysis process.

Figure 2 showed that ORP sharply decreased to about 150 mV when the simulated seawater flowed through the electrolysis unit in 30 min. When the effluent of electrolysis cell flowed into buffer tank mixing with water flow directly coming from breeding tanks, ORP remained at around 275 mV. Then the buffer pool water flowed into the activated carbon filter. As a result of the adsorption, ORP decreased to some extent, which was similar to that in the original breeding pool. In general, during the 3 h of electrolysis action, ORP almost remained at around 225 mV in the breeding tanks. Therefore, in the process of electrolysis action, ORP basically kept stable. Generally, ORP has been used as the monitoring and controlling parameter in many wastewater treatment plants, especially in chemical treatment and biological treatment units in Taiwan^31^. And ORP also is the most basic parameter used to characterize the redox properties of seawater in marine aquaculture. It can be used to determine the stability of the redox couple in each form^32^. At the same time, a variety of chemical reactions, especially the various biochemical reactions that are caused by the cells of organism, are completed in a specific range of oxidation-reduction potential. Thus, it is important to maintain the stable ORP.

**Figure 3:**
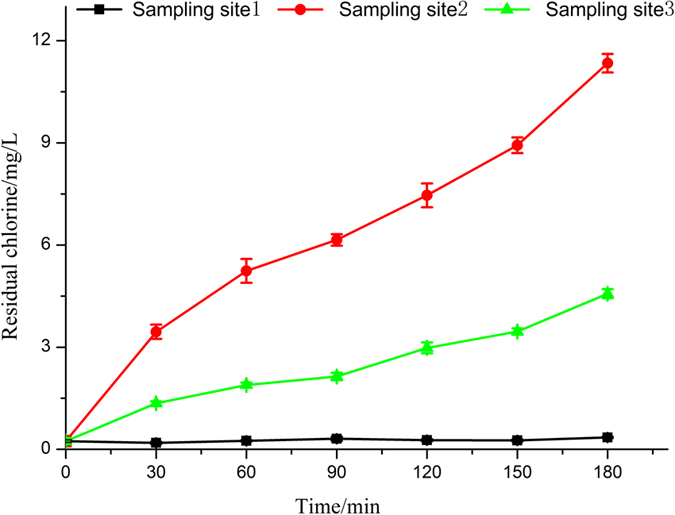
Change in residual chlorine of simulated seawater during electrolysis process.

The production of residual chlorine during the electrolytic process at different sampling locations was shown in Fig. 3. Obviously, when the simulated seawater flowed through the combined electrolysis cell, the concentration of residual chlorine increased rapidly, especially in the first 30 minutes. And then, the concentration showed a steady ascending tendency, up to 11.34 mg/L after 3 h electrolysis. When the effluent of electrolysis cell flowed into buffer tank mixing with water flow directly coming from breeding tanks, the concentration of residual chlorine increased from 0.24 mg/L to 4.6 mg/L. Then the buffer pool water flowed into the activated carbon filter. Because of the adsorption and aeration, residual chlorine quickly dropped to about 0.2 mg/L. As a whole, the concentration of residual chlorine remained at around 0.1~0.5 mg/L in the tanks during the process of electrolysis, less than median lethal concentration (LC_50_) of tilapia. During the electrochemical process, excessive residual chlorine will cause damage to the gills of fish, which can hinder the exchange of dissolved oxygen in fish gills and water^33^. In addition, the residual chlorine will go through the gills tissue and infiltrate into the blood to oxidize the reducing hemoglobin carrying oxygen in blood. And it may also inhibit the enzyme activity of methemoglobin, resulting in a decrease of carrying oxygen capacity in blood^34^. Therefore, in order to ensure the safety of fish in the tanks, the amount of the residual chlorine was controlled by the activated carbon filter.

**Figure 4:**
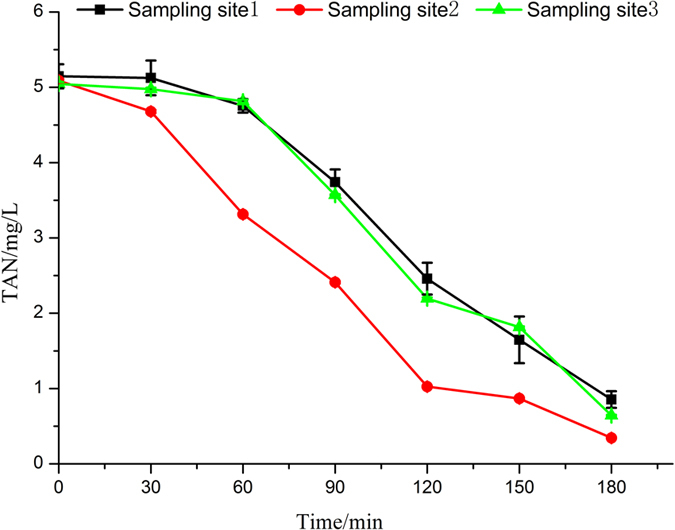
Change in TAN concentration of simulated seawater during electrolysis process.

As is shown in Fig. 4, with the simulated seawater flowing through the electrolysis unit, the removal of TAN was accelerated owing to the generation of hydroxyl radical and chlorine radical and the direct electrochemical oxidation in the process of combined effect of electrolysis and ultraviolet. TAN concentration decreased from 5.089 mg/L to 1.026 mg/L quickly within 120 min. Nevertheless, the concentration of TAN had little change in the buffer pool and tanks within 60 min. And then that began to decline rapidly. The concentration of TAN was reduced to about 0.7 mg/L after 180 min. On the basis of one-way ANOVA for the TAN concentration in seawater at different time points within 180 min, the results showed that the concentration of TAN had no significant differences in the buffer pool and breeding tanks, but higher than that of the electrolysis unit effluent. It had been proved that NH_4_^+^ could not be oxidized by direct electrochemical oxidation and only molecular form of ammonia (NH_3_) could be directly electro-oxidized in acid solutions^35^. Therefore, this phenomenon could be concluded that the reaction between active chlorine and UV light played a dominant role in the combined electrolysis cell. And in the high concentration of active chlorine/UV system, active chlorine would indeed dissociate to ·OH and ·Cl radicals under UV irradiation and the photogenerated radicals should be responsible for the accelerating effect on ammonia degradation in the electrochemical process combined with UV light^35^,^36^. Because the photogenerated radicals were very short-lived, indirectly electrochemical oxidation was mainly caused by active chlorine in the buffer pool. So the removal of TAN in the buffer pool was weaker than that in the combined electrolysis cell. In general, the TAN concentration of 5 mg/L could be reduced to 1 mg/L in the culture pond after 3 h, which would not affect the normal growth of tilapia.

**Figure 5:**
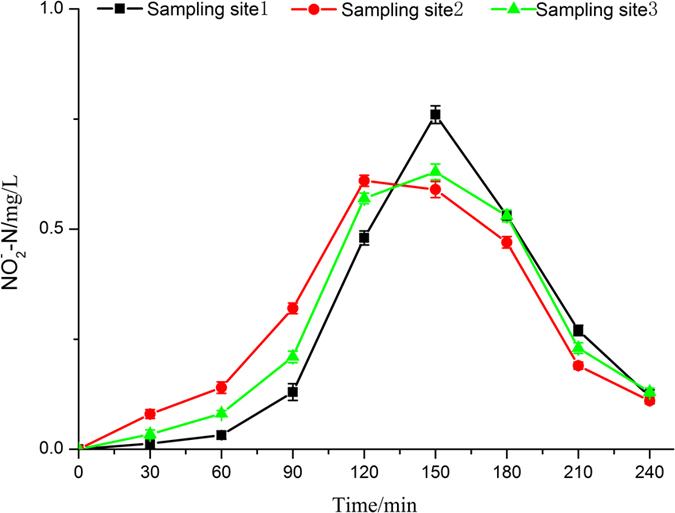
Change in nitrite of simulated seawater during electrolysis process.

Regarding Fig. 5, nitrite concentration gradually increased with electrolysis time going on when cultivating water flowed through electrolysis unit. The nitrite concentration reached maximum at 150 min, approximately about 0.76 mg/L. Soon afterwards, nitrite concentration began to decline rapidly, dropping down to 0.12 mg/L at 240 min. In this process, the change trend of nitrite concentration in the breeding tanks and buffer pool was similar to that in the electrolysis unit. At the same time, the nitrite concentration in breeding tanks was lower than that in the electrolysis cell and buffer pool within 150 minutes, which was consistent with PérezG’s research conclusion^37^. At the beginning of the experiment, active chlorine was produced in the electrochemical process according to Eqs (2 and 3). Owing to the high concentration of TAN at this point, it would be oxidized by the active chlorine through indirect oxidation reaction, as it is indicated by Eqs (6 and 9) 37. With the increase of the concentration of nitrate, there was denitrification of part nitrate during the electrolysis process, as is shown in Eq. (10) 37. And then the concentration of nitrite reached maximum at about 150 min. Alone the experimental time, nitrite was reduced leading to the formation of nitrate according to Eq. (11) 37. This may be the reason for the trend of concentration of nitrite, which is shown in Fig. 5.


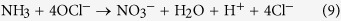



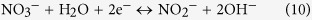



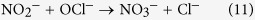


**Figure 6:**
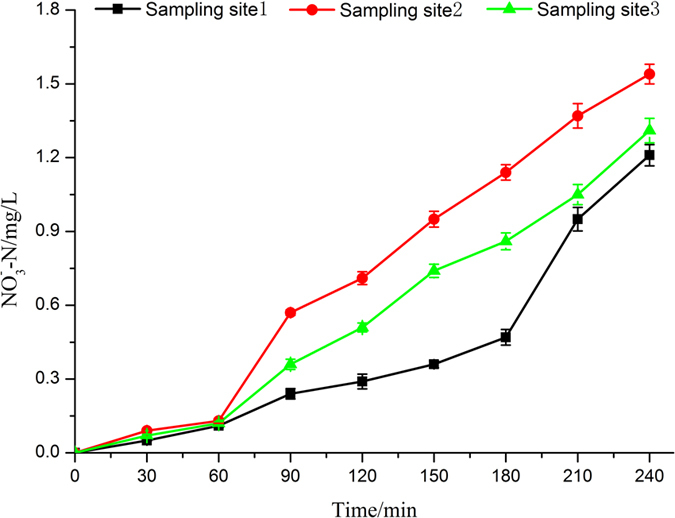
Change in nitrate of simulated seawater during electrolysis process.

The results from Fig. 6 indicated that the concentration of nitrate rose by only a meagre amount and remained relatively stable within 60 min in the three sampling points during the water treatment process of micro current electrolysis combined with UV-light. The change of nitrate concentrations had little difference among the three sampling sites. With regard to these issues, maybe because the limited active chlorine is produced in this process. Over time, nitrate concentration increase rapidly by different rates among the three sampling points, the highest concentration in the electrolysis unit, then came that in the buffer pool, with the lowest in the tanks. This may due to the increase of active chlorine concentration and the synergistic effects of micro current electrolysis combined with UV-light. Finally, nitrate concentration reached at 1.23 mg/L in breeding tanks at 240 min. The toxicity of nitrate is lower than that of ammonia or nitrite. Nitrate, the end product of nitrification, is relatively nontoxic except at very high concentrations (over 300 mg/L). Usually nitrate does not build up to a very high concentration if some daily exchange (5 to 10 percent) with fresh water is part of the management routine^38^.

### Small pilot micro current electrolysis combined UV-light RAS experiment

**Figure 7:**
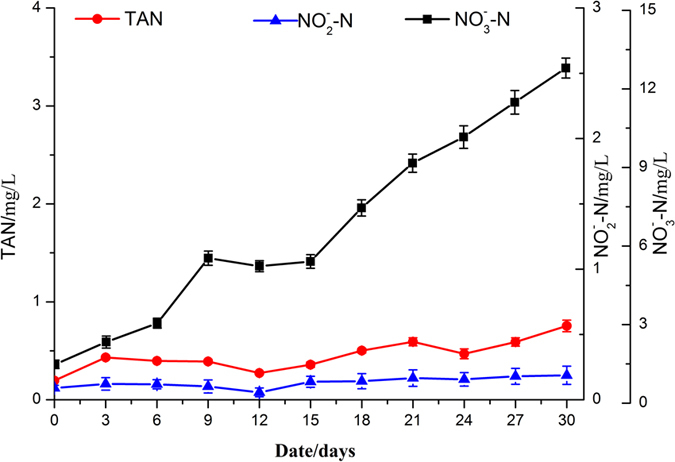
Change in nitrogen species concentrations in the fish tank during the growth experiment.

During the culture period, temperature and dissolved oxygen (DO) in water were in optimal condition for fish culture which were ranged from 25–29 °C and 6.0~9.0 mg/L. The concentrations of TAN, nitrite and nitrate were presented in Fig. 7. The actual concentration of TAN remained stable at below 1 mg/L throughout the experiment. At the same time, nitrite concentration throughout the culture period appeared to be lower than 0.2 mg/L. And nitrate accumulation was observed which was followed by a slight decline during 9–15 days. This change probably related to the decrease of the TAN during the period. Altogether, the concentration of nitrate continuously rose reached at 12.79 mg/L. In the previous works describing the water quality in the process of culturing tilapia, ZH Wang reported that the concentrations of TAN and nitrite kept in the range of 0.36~0.52 mg/L and 0.02~0.06 mg/L in a conventional RAS for over a month^39^. It seemed that the concentrations of TAN and nitrite were slightly lower than that in this research. Because there is a difference in stocking density between the two study. The stocking density in this research was more than double that in the conventional RAS which was 6 kg/m^3^. Besides, WIDANARNI pointed out that the total ammonia nitrogen concentrations in biofloc technology (BFT) treatments, regardless the density, remained stable at below 1.1 mg/L throughout the culture period of 14 weeks^40^. BFT is an aquaculture system which focused on a more efficient use of nutrient input with limited or zero water exchange. And the nitrite concentration appeared to be fluctuated during the culture period. Meanwhile, in the research made by Ben Asher and Lahav, N-allylthiourea, a nitrification inhibitor was added to the fish (gilthead seabream) tank twice a week in order to minimize the formation of unwanted NO_2_^−^/NO_3_^−^ during the electro-oxidation process. They concluded nitrite concentrations, resulting from both partial nitrification and the oxidation of nitrite in the electrolysis step, were relatively stable (3.9 ± 1.1 mg N/L)^41^. In our research nitrite concentrations maintained below 0.2 mg/L without addition of nitrification inhibitor. Perhaps the photogenerated radicals was responsible for the promotion effect on nitrite removal during the electrochemical process combined with UV light. In addition, nitrite was more easily oxidized to nitrate than TAN^19^,^41^. So there was relatively low concentration of nitrite in this study. Thus, it can be stated with reasonable certainty that the TAN and nitrite were converted to nitrate by the electrochemical method in the innovative RAS that combined micro current electrolysis with UV-light on water treatment, which make sure tilapia fish were comfortable in the conditions applied in the experiment.

**Figure 8:**
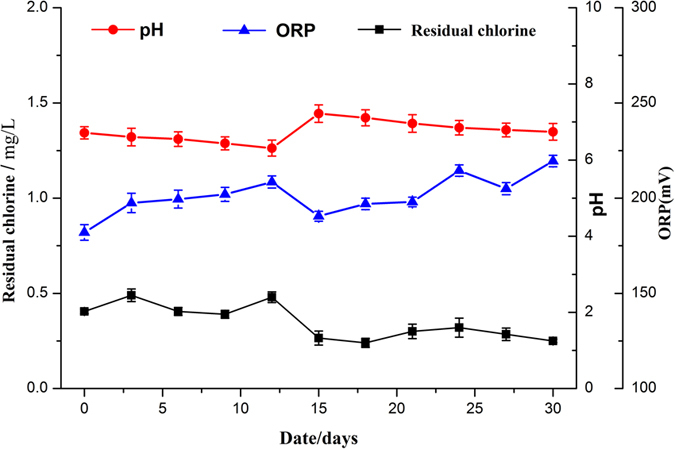
Change in pH, ORP and residual chlorine in the fish tank during the growth experiment.

Figure 8 shows the value of pH, ORP and residual chlorine in the fish tank during culture period. The concentration of residual chlorine in pond within 15 days showed a relatively stable level at a range of 0.25~0.5 mg/L, and then decreased gradually, eventually reached below 0.3 mg/L, this may be due to the increase of organic matter accumulated in activated carbon filter, which consumed part of residual chlorine. In the preliminary fundamental research ORP basically kept stable and almost remained at around 225 mV in the breeding tanks. But in the small pilot scale process the picture was different, the ORP of aquaculture water fluctuated slightly in the range of 190~240 mV, and did not show a certain regularity. This probably due to a certain organic matter concentration entrapped in the electrolysis unit during the electrochemical process, which influenced the generation of available chlorine. As is shown in Fig. 8, the change rule of ORP was almost in line with that of residual chlorine. In addition, the pH maintained a downward trend at the beginning, and increased suddenly from 6.40 to 7.32 on 12^th^ days, which was due to adding 80 g baking soda to adjust pH according to method which was mentioned in the fundamental research. It is considered ideal for tilapia within the pH range between 7 and 8^42^,^43^. This suggests that the pH correction by adding baking soda has little influence on tilapia during the experiment. Therefore medical stone or baking soda needed to add in the buffer pool during the electro-oxidation process to adjust pH, otherwise the pH decrease would affect the growth of tilapia.

**Figure 9:**
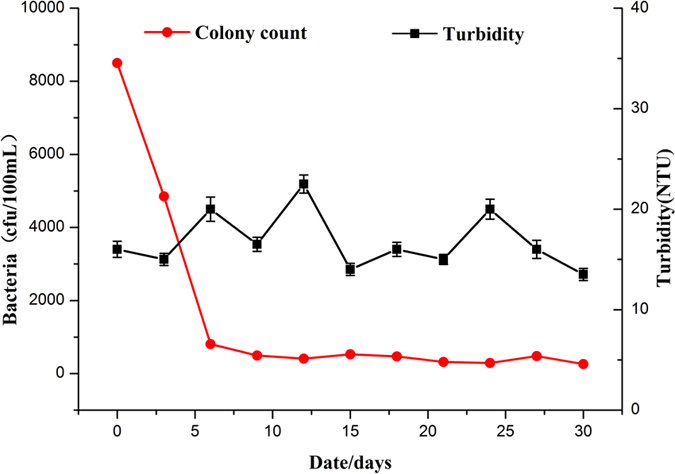
Change in turbidity and total bacteria count in the fish tank during the growth experiment.

Electrochemical treatment of water has shown great potential for disinfection. And it is known to inactivate a wide variety of microorganisms from bacteria to viruses and algae^44^,^45^,^46^. One advantage of electrochemical disinfection is that the damage produced to the bacterial cells is more severe than that produced by pure chemical disinfection with chlorine^47^. In this study, disinfection experiments were carried out by electrochemical treatment of aquaculture water with *E. coli.* Figure 9 shows the inactivation of *E. coli* during the electrolysis process. The initial total bacteria count in the fish tank was 8500cfu/100 mL, and fell quickly to 810cfu/100 mL during the first 5 days, hereafter the total bacteria count remained lower than 500cfu/100 mL, namely under the aquaculture water quality standard of NY5051-2001. Due to the existence of activated carbon filter, the turbidity of aquaculture water always kept in the range of 14–22NTU. On the one hand lower turbidity improved the electrolysis efficiency, on the other hand it could strengthen the bactericidal effect of ultraviolet lamp. During the growth experiment the turbidity of aquaculture water fluctuated slightly, the bactericidal effect of micro electrolysis combined with ultraviolet system was almost unaffected. Compared with the removal of TAN and nitrite, less time was required for inactivation of microorganisms during the process of electrolysis combined with UV-light, which meant no extra energy was needed to be consumed for disinfection.

### Fish performance

**Figure 10:**
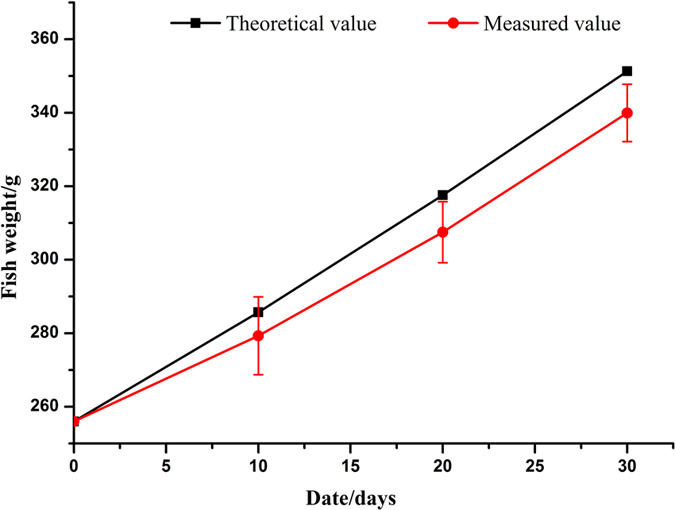
Change in weight of Gift during the growth experiment.

Figure 10 shows average weights of the fish during the growth period, as compared with the growth potential of tilapia at 28 °C, determined using eq. (12)^48^:


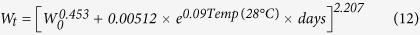


where *W*_*t*_ (g) is the fish weight after *t* days of growth and *W*_*0*_ is the initial fish weight (g).

During the 30 days of experiment, water quality parameters remained stable, tilapia weight maintained sustained growth at a rate similar to the theoretical potential growth rate. Tilapia weight reached at 339.3 ± 10 g at the end of culture period, lower than the theoretical value (351 g as calculated) of 12 g. This may be due to the water temperature could not always stay at 28 °C, which affected the food consumption of tilapia. Despite not being a very long growth period, periodical observations proved the fish to be in excellent condition throughout the growth period.

Various biochemical indicators in serum reflect health status and metabolic function of the tissue faster than the representational change of body. In order to explore the effect of micro current electrolysis combined with UV-light on cultured species for water treatment, the activity of carbonic anhydrase, transaminase and glutamic dehydrogenase in tilapia serum were determined. The results are shown in Table 1.

Carbonic anhydrase is one of the core enzyme in organism, which involves in osmoregulation, ionic regulation, acid-base regulation and other physiological and biochemical process. Whether the regulation of hypertonic or hypotonic, the carbonic anhydrase activity of osteichthyes showed increasing trend^49^. In the course of the experiment, carbonic anhydrase activity increased from 23.97 U/L to 40.29 U/L and improved 67.11%. But it is still in the range of 0–90 U/L. The main reason that caused the rise of carbonic anhydrase activity was the frequent change of makeup water in the breeding process, which caused fluctuations in ion concentration of aquaculture water and changed the osmotic pressure in the tilapia body. Meanwhile, the increase of carbonic anhydrase activity will enhance the ability of automatic regulation acid-base balance of fish humoral.

Transaminase is an essential catalyst in the metabolic process. When the hepatocellular are damaged, the aminotransferase will be released into the blood, so that the serum aminotransferase will increase. Transaminase elevation is a very common situation. Because of the high sensitivity of transaminase, many factors will cause the fluctuation of normal transaminase values. Different inspection time within one day, transaminase measurement results are likely to be different^50^. By sampling in various regions of the country, Lihong Wang determined the activity changes of tilapia transaminase and found that the scope of activity ranged from 80 U/L to 1300 U/L^51^. In the process of the experiment, transaminase activity increased 35.48%. And it always kept in the scope of 147.35~226.33 U/L. Further work is therefore required to explore whether the damage of hepatocellular caused the rise of transaminase activity in the following research.

Glutamate dehydrogenase (GHD), one of mitochondrial enzyme, plays an important role in the metabolism of amino acids. The activity of glutamate dehydrogenase is influenced by water temperature and other conditions^52^. During the process of the experiment, the glutamate dehydrogenase activity increased from 183.65 U/L to 273.8 U/L, improved by 46.53%. And it still remained in the normal range of 100~400 U/L. The activity changes of glutamate dehydrogenase can be influenced by many factors, which still need to be further explored.

## Conclusion

This paper discusses an approach of micro current electrolysis combined with UV-light on water treatment in saline RAS, which successfully applied within the operation of both the fundamental research and small pilot scale experiment. The conclusions can be drawn as follows:

1. Based on the electrolysis experiment in the fundamental research, TAN was removed effectively in simulated water. And water quality parameters remained relatively stable. This ensured the smooth development of the small pilot scale experiment.
2. The technology combined micro electrolysis with UV-light could effectively remove the TAN and keep the concentration of nitrite at a low level in aquaculture water, but at the same time, it caused the increase of nitrate concentration. Due to the adsorption of activated carbon filter, the concentration of residual chlorine in water decreased gradually with culture time prolonging. And pH had followed the same trend, which need to take regular adjustment. In addition, the ORP of the water had small amplitude fluctuations in the electrolysis process, which would not have an impact on fish. Because of residual chlorine, microorganisms could be killed quickly. And the turbidity fluctuations had no significant effect on the bactericidal effect.
3. During the practical breeding process, tilapia weight maintained sustained growth. Compared with the theoretical growth, there were no significant differences. The activity determination of glutamate dehydrogenase, alanine aminotransferase and carbonic anhydrase showed that enzyme activity slightly increased, but all values were in the normal range. It has been proved that there are technical feasibilities which combine micro current electrolysis with UV-light on water treatment in saline RAS.

## Materials and Methods

All experimental protocols were approved by the committee of the Care and Use of Animals of the Zhejiang University. The methods were carried out in strict accordance with the guidelines of the Association for the Study of Animal Behavior Use of the Zhejiang University.

### Experimental materials and set up

Genetic Improvement of Farmed Tilapia (GIFT, Guangxi, China) was chosen as the proof-of-concept fish species due to its relative tolerance to dissolved NH_3_ and CO_2_ concentrations. For rearing the tilapia, two different culture systems were used. One is a traditional RAS with a MBBR bio-filter that was applied to domestication of the tilapia, the other is an innovative RAS which combined micro current electrolysis with UV-light on water treatment. Each culture system with about 700 L water contained 2 replicate tanks. The volume of the water was about 300 L in each breeding tanks. The tilapia was domesticated by adding artificial salt (Zhejiang, China) for 30 days in the traditional RAS. The salinity increased 1‰ per day. During the 30 days domestication period, the system was operated with 5% daily make up water exchange. Feeding rate was accord to 1% of the fish weight. The water quality indicators are as follows: water temperature 23~27 °C, pH 7.5~7.9, dissolved oxygen (DO) 6.0~9.0 mg/L.

**Figure 11:**
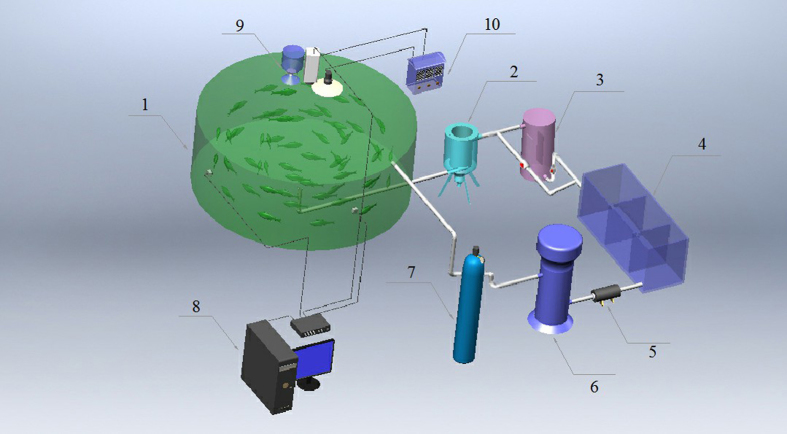
Experimental Setup. 1. Culture tank; 2. Solid-liquid separator; 3. Combined electrolysis cell; 4. Buffer pool; 5. Pump; 6. Protein separator; 7. Activated carbon filter; 8. Computer; 9. Monitoring system; 10. Controller.

**Figure 12:**
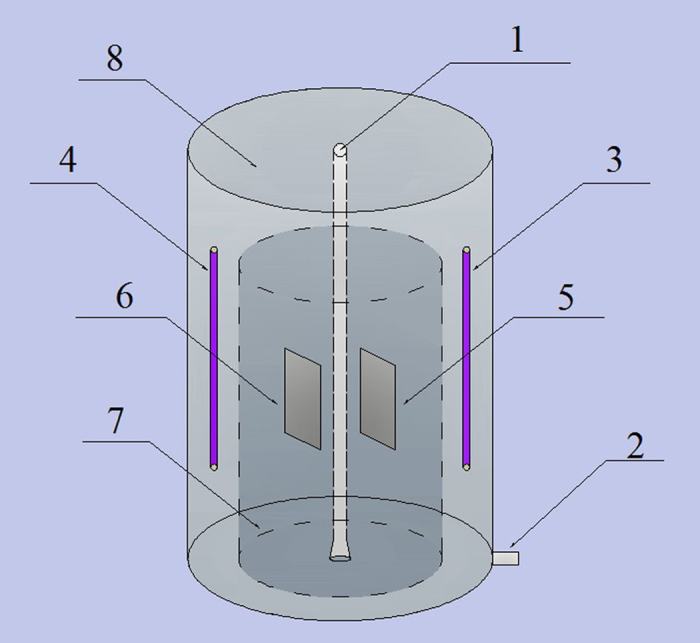
The unit of micro current electrolysis combined UV-light. 1. Inlet; 2. Outlet; 3. Ultraviolet lamp (1); 4. Ultraviolet lamp (2); 5. Anode; 6. Cathode; 7. Inner tank; 8. Outer tank.

The recirculating aquaculture system which based on electrolysis combined with ultraviolet light is shown as Fig. 11. Technical details of all the equipment are as follows. The size of fiberglass culture pond is Φ800 × 750 mm, the flow of solid-liquid separator was 1000 L/h. The structure of combined electrolysis cell was shown as Fig. 12, the capacity of ultraviolet lamp is 16 W, the size of Ti-Pt electrode is 25 × 15 cm, the size of buffer pool is 1000 × 300 × 500 mm, and the height of activated carbon filter is 1250 mm, which the height of quart sand is 150 mm and the height of activated carbon is 900 mm.

### Fundamental research

In order to determine the electrolysis conditions and system security during the practical culture experiment, the change of water quality parameters and the removal efficiency of TAN were researched in the process of electrolyzing the simulated seawater. Before breeding experiment was carried out in the innovative RAS, 10 g of ammonium chloride was added into the simulated seawater to reach a desired initial concentration of TAN at 5 mg/L. In the process of industrial RAS, a certain amount of fresh water was added into the tanks to adjust the water temperature before seawater was introduced into the breeding system. After that, the seawater salinity decreased to around 25‰. Therefore, simulated seawater was prepared by adding the artificial salt into tap water to reach the salinity about 25‰ during the fundamental research.

#### Electrolysis conditions

In the no-diaphragm electrolysis system, the amount of residual chlorine is a fixed value when electrolytic tank go through the power of 1 A.h^53^. The electrolysis conditions were calculated according to the data of previous experiment (Supplementary Information) and faraday’s law of electrolysis. The electrolysis voltage and current were 15 V and 2.5 A, respectively. During the electrolysis process, large percentage (2/3) of the effluent of the breeding tanks flowed into the buffer pool. The other part (1/3) flowed through the combined electrolysis unit and then flowed into the buffer pool. The flow ratio of these two parts (the effluent of electrolysis cell and water flow directly coming from breeding tanks) was 1:2. Electrolysis time was 3 h.

#### Sampling of water and determination of water quality parameters

During the experimental period, water samples were taken regularly from three sampling points using a pipette for analysis of different parameters. Three sampling points were set up in the system. Sampling point 1 was set in the two breeding tanks. The position of sampling point 2 was in the outlet of the combined electrolysis unit. Another sampling point was in the buffer pool. Sampling interval was 30 min. Total ammonia nitrogen (TAN), NO_3_^−^-N and NO_2_^−^-N concentrations were analyzed using a Cary 60 UV-vis spectrophotometer (Agilent Technologies, America) according to standard methods (SEPA, 2002). The pH and oxidation reduction potential (ORP) were measured with pH/ORP/Conductivity meter (Seven-Multi, Mettler-Toledo Instruments Co., Ltd., China). Residual chlorine was analyzed using a pocket chlorimeter (RC-3F, Dai li co., Ltd) according to DPD (N,N-diethyl-p-phenylenediamine) method.

### Small pilot micro current electrolysis combined UV-light RAS experiment

After electrolysis of simulated seawater, make up water exchange transferred to the system. The concentrations of TAN and nitrite maintained below 0.2 mg/L. pH ranged from 7.30 to 7.60 at a stable state. Make up water supply was 5% per day. The salinity was kept at about 25‰ by adding the artificial salt. After 24 h oxygen aeration, the tilapia which has been domesticated was introduced into the innovative RAS that combined micro current electrolysis with UV-light on water treatment. 42 tilapia fish, with an initial average weight of 250 ± 5 g, were stocked in two ponds making for an initial fish density of 13 kg/m^3^. After 3 days of adaptation period, the electrolysis experiment was carried out. Daily feeding time is 9:00 am, 13:00 pm and 18:00 pm. Feeding rate was accord to 3% of the fish weight. During the experiment, the fish feed should be increased according to the theoretical increment of body weight. The bait that was not eaten up should be scooped out and weighed in time.

#### Electrolysis conditions

Compared with the electrolysis experiment in the fundamental research, the unit which combined micro current electrolysis with UV-light run 5 h (9:00~14:00). And other electrolysis conditions remain consistent.

#### Determination of water quality parameters and weighing of fish

Using the identical sampling method, residual chlorine, pH, ORP and fish tank total ammonia nitrogen (TAN), NO_3_^−^-N and NO_2_^−^-N concentrations were measured daily by using the same test methods which were described in the determination of water quality parameters. Daily sampling time is at 9:00 am due to higher accumulation of water pollution substance. And turbidity was measured with a turbidimeter. Membrane filtration method was used for detection of microorganism. Five tilapia were randomly collected from the tanks for calculating the average weight every 10 days.

#### Determination of enzyme activity

After the 30 days of breeding experiment, eight fish were randomly selected from the two sets of culture systems for blood samples to verify the impact on enzymatic activity. Four of them were from the traditional RAS in which the tilapia have been domesticated. The others came from the innovative RAS. In the electrolysis process, residual chlorine make a great difference on enzyme of gill tissue. So the blood samples obtained from branchial artery using 10 ml disposable syringes. During the blood sampling, the tilapia was wrapped in wet towel. Blood samples were centrifuged for 5 min at the rate of 2000 r/min. After standing and layering process, supernatant was cryopreserved until processing. And then, the activity of glutamate dehydrogenase, transaminase and carbonic anhydrase were determinated by the corresponding enzymatic kit.

## Additional Information

**How to cite this article**: Ye, Z. *et al*. Synergistic Effects of Micro-electrolysis-Photocatalysis on Water Treatment and Fish Performance in Saline Recirculating Aquaculture System. *Sci. Rep.* **7**, 45066; doi: 10.1038/srep45066 (2017).

**Publisher's note:** Springer Nature remains neutral with regard to jurisdictional claims in published maps and institutional affiliations.

## Supplementary Material

Supplementary Information

## Figures and Tables

**Table 1:** The effect of enzymatic activity during electrochemical process combined UV-light (U/L).

	Control group				experimental group			
	1#	2#	3#	4#	5#	6#	7#	8#
carbonic anhydrase	25.20 ± 0.15	22.70 ± 1.24	24.01 ± 0.79	23.97 ± 0.47	42.25 ± 0.91	41.03 ± 1.22	36.58 ± 0.48	41.31 ± 1.05
transaminase	147.35 ± 8.19	171.64 ± 7.70	156.53 ± 5.31	158.51 ± 3.17	202.40 ± 5.60	198.80 ± 7.50	226.33 ± 3.10	205.82 ± 4.44
glutamate dehydrogenase	154.26 ± 6.48	216.25 ± 3.17	180.54 ± 1.59	183.65 ± 4.21	293.51 ± 12.46	261.51 ± 6.26	305.59 ± 3.12	234.59 ± 3.34

## References

[b1] Hao X., Liu Y. & Ling Q. Acute Toxicity test of ammonia nitrogen and effects of ammonia-N stress on the ultrastructure of gill and liver of *Misgurnus anguillicaudatus*. Journal of Hydroecology 33, 101–107 (2012).

[b2] Chris G. J., Van, B., Jan P. S., Sven W. & Carsten S. The chronic effect of nitrate on production performance and health status of juvenile turbot (*Psetta maxima*). Aquaculture 326–329, 163–167 (2012).

[b3] Sun G., Tang J. & Zhong X. Toxicity research of ammonia nitrogen and Nitrite Nitrogen to *penaeus vannamei*. Journal of Aquaculture 1, 22–24 (2002).

[b4] Shan B., Wang X., Zhao Z. & Hu H. Advances in research of wastewater treatment technology of marine aquaculture plant. Marine science 10, 36–38 (2002).

[b5] Wang W. Research on microbial biofilm of biological stuffings and effect of water treatment in marine recirculating aquaculture. Ocean University of China (2012).

[b6] Shi F. . Pilot study on ammonium removal efficiency and nitrifying bacteria variety in marine biofilter charged with bamboo substrate. Process in fishery science 31, 92–95 (2009).

[b7] Gendel Y. & Lahav O. A novel approach for ammonia removal from fresh-water recirculated aquaculture systems, comprising ion exchange and electrochemical regeneration. Acquacultural Engineering 52, 27–38 (2013).

[b8] Zhou Y. . Effect of ozone dosing location changed on water quality in enclosed recirculating aquaculture system for tongue sole (*Cynoglossus semilaevis*). Fishery Modernization 39, 5–9 (2012).

[b9] Rajeshwar K., Ibanez J. G. & Swain G. M. Electrochemistry and the environment. J A ppl Electrochem. 24, 1077–1091 (1994).

[b10] Zang W. . The technique and mode of regulating-controlling circulation water for indoor industrial culture of *Penaeus vannamei*. Journal of fisheries of China 5, 749–757 (2008).

[b11] Díaz V., Ibáñez R., Gómez P., Urtiaga A. M. & Ortiz I. Kinetics of electro-oxidation of ammonia-N, nitrites and COD from a recirculating aquaculture saline water system using BDD anodes. Water Research 45, 125–134 (2011). 20832837 10.1016/j.watres.2010.08.020

[b12] Basha C. A., Chithra E. & Sripriyalakshmi N. K. Electro-degradation and biological oxidation of non-biodegradable organic contaminants. Chemical engineering journal 149, 25–34 (2009).

[b13] Ou Y., Shang X., Wang X. & Kong H. Study of total ammonia nitrogen removal in swine wastewater by electrochemical oxidation process. Technology of water treatment 06, 111–115 (2010).

[b14] Daghrir R., Drogui P. & Tshibangu J. Efficient treatment of domestic wastewater by electrochemical oxidation process using bored doped diamond anode. Separation and Purification Technology 131, 79–83 (2014).

[b15] Wang C. T., Chou W. L., Kuo Y. M. & Chang F. L. Paired removal of color and COD from textile dyeing wastewater by simultaneous anodic and indirect cathodic oxidation. Journal of hazardous materials 169, 16–22 (2009). 19362772 10.1016/j.jhazmat.2009.03.054

[b16] Deng Y. & Englehardt J. D. Electrochemical oxidation for landfill leachate treatment. Waste management 27, 380–388 (2007). 16632340 10.1016/j.wasman.2006.02.004

[b17] Costa C. R., Botta C. M., Espindola E. L. & Olivi P. Electrochemical treatment of tannery wastewater using DSA^®^ electrodes. Journal of hazardous materials 153, 616–627 (2008). 17931769 10.1016/j.jhazmat.2007.09.005

[b18] Kim K. W., Kim Y. J., Kim I. T., Park G. I. & Lee I. H. The electrolytic decomposition mechanism of ammonia to nitrogen at an IrO_2_ anode. Electrochimica Acta 50, 4356–4364 (2005).

[b19] Lin S. H. & Wu C. L. Electrochemical removal of nitrite and ammonia for aquaculture. Water research 30, 715–721 (1996).

[b20] Vooys A. C. A., Santen R. A. & Veen J. A. R. Electrocatalytic reduction of NO_3_^−^on palladium/copper electrodes. Journal of Molecular Catalysis A: Chemical 54, 203–215 (2000).

[b21] Chiang L., Chang J. & Wen T. Indirect oxidation effect in electrochemical oxidation treatment of landfill leachate. Water Research 29, 671–678 (1995).

[b22] Vanlangendonck Y., Corbisier D. & Van L. A. Influence of operating conditions on the ammonia electro-oxidation rate in wastewaters from power plants. Water Research 39, 3028–3034 (2005). 16000211 10.1016/j.watres.2005.05.013

[b23] Gendel Y. & Lahav O. Revealing the mechanism of indirect ammonia electro-oxidation. Electrochimica Acta 63, 209–219 (2012).

[b24] Feng Y., Smith D. W. & Bolton J. R. Photolysis of aqueous free chlorine species (HOCl and OCl^−^) with 254 nm ultraviolet light. Journal of Environmental Engineering and Science 6, 277–284 (2007).

[b25] Jin J., Eldin M. G. & Bolton J. R. Assessment of the UV/Chlorine process as an advanced oxidation process. Water Research 45, 1890–1896 (2011). 21211812 10.1016/j.watres.2010.12.008

[b26] Guo H. Time-dependent quantum dynamical study of the photo dissociation of HOCl. Journal of Physical Chemistry 97, 2602–2608 (1993).

[b27] Molina M. J., Ishiwata T. & Molina L. T. Production of OH from photolysis of HOCl at 307–309 nm. Journal Physical Chemistry 84, 821–826 (1980).

[b28] Olsson B. E. R., Hallquist M., Ljungström E. & Davidsson J. A kinetic study of chlorine radical reactions with ketones by laser photolysis technique. International Journal of Chemical Kinetics 29, 195–201(1997).

[b29] Chuang Q. I. . Study of pH regulation technology in the recirculating seawater of aquaculture system. Journal of Biology 29, 39–42 (2012).

[b30] Juan L. I., Zhang P. Y., Gao Y., Song X. G. & Dong J. H. Overview of Maifanshi: its physi-chemical properties and nutritious function in drinking water. Environ. Sci. Technol. 31, 63–333 (2008).

[b31] Chang C. N., Ma Y. S. & Lo C. W. Application of oxidation-reduction potentials as a controlling parameter in waste activated sludge hydrolysis. Chemical Engineering Journal 90, 273–281 (2002).

[b32] Saby S., Djafer M. & Chen G. H. Effect of low ORP in anoxic sludge zone on excess sludge production in oxic-settling-anoxic activated sludge process. Water Research 37, 11–20 (2003). 12465783 10.1016/s0043-1354(02)00253-1

[b33] Mitz S. V. & Giesy J. P. Sewage effluent biomonitoring: I. Survival, growth, and histopathological effects in channel catfish. Ecotoxicology and Environmental Safety 10, 22–39 (1985). 4029059 10.1016/0147-6513(85)90004-1

[b34] Zeitoun I. H. The effect of chlorine toxicity on certain blood parameters of adult rainbow trout (*Salmo gairdneri*). Environmental Biology of Fishes 1, 189–195 (1977).

[b35] Xiao S. H., Qu J. H., Xu Z., Liu H. J. & Wan D. J. Electrochemical process combined with UV light irradiation for synergistic degradation of ammonia in chloride-containing solutions. Water Research 43, 1432–40 (2009). 19135227 10.1016/j.watres.2008.12.023

[b36] Wu Z. . The Study of the removal of ammonia in RAS by using electrochemical process combined with UV irradiation. Transactions of the Chinese Society for Agricultural Machinery (2016).

[b37] Pérez G., Ibáñez R., Urtiaga A. M. & Ortiz I. Kinetic study of the simultaneous electrochemical removal of aqueous nitrogen compounds using BDD electrodes. Chemical Engineering Journal 197, 475–482 (2012).

[b38] Masser M. P., Rakocy J. & Losordo T. M. Recirculating aquaculture tank production systems: Management of recirculating systems. SRAC Publication No. 452 (1999).

[b39] Wang Z. & Liu H. Nitrogen budget and water quality of tilapia in recirculating aquaculture system. Fishery Modernization 5, 12–16 (2011).

[b40] Widanarni Ekasari J. & Maryam S. Evaluation of biofloc technology application on water quality and production performance of red tilapia *Oreochromis sp.* cultured at different stocking densities. HAYATI Journal of Biosciences 19, 73–80 (2012).

[b41] Ben-Asher R. & Lahav O. Electrooxidation for simultaneous ammonia control and disinfection in seawater recirculating aquaculture systems. Aquacultural Engineering 72, 77–87 (2016).

[b42] El-Sayed A. E. M. Tilapia culture. CAB International, Oxfordshire, UK (2006).

[b43] El-Sherif M. S. & El-Feky A. M. I. Performance of Nile tilapia (*Oreochromis niloticus*) fingerlings. I. Effect of pH. International Journal of Agriculture & Biology 11, 297–300 (2009).

[b44] Tanaka T. . Electrochemical disinfection of fish pathogens in seawater without the production of a lethal concentration of chlorine using a flow reactor. Journal of Bioscience & Bioengineering 116, 480–484 (2013). 23648105 10.1016/j.jbiosc.2013.04.013

[b45] Feng C. . Water disinfection by electrochemical treatment. Bioresource Technology 94, 21–25 (2004). 15081482 10.1016/j.biortech.2003.11.021

[b46] Liang W., Qu J., Chen L., Liu H. & Lei P. Inactivation of *Microcystis aeruginosa* by continuous electrochemical cycling process in tube using Ti/RuO_2_ electrodes. Environ. Sci. Technol. 39, 4633–4639 (2005). 16047803 10.1021/es048382m

[b47] Wang Y., Claeys L., Ha D. V. D., Verstraete W. & Boon N. Effects of chemically and electrochemically dosed chlorine on *Escherichia coli* and *Legionella beliardensis* assessed by flow cytometry. Applied microbiology and biotechnology 87, 331–341 (2010). 20352423 10.1007/s00253-010-2526-2

[b48] Lupatsch I. Predicting growth, feed intake and waste production of intensively reared tilapia based on nutritional bioenergetics. *In: Proceedings of the Seventh International Conference on Recirculating Aquaculture, Roanoke, Virginia*, pp. 306–314.

[b49] He F., Xiang J., Li C., Li Z. & Chen K. Preliminary study on the effect of water temperature on hematology indices of Rainbow Trout. Acta Hydrobiologica Sinica. 3, 363–369 (2007).

[b50] Esbaugh A. J. . Cytoplasmic carbonic anhydrase isozymes in rainbow trout *Oncorhynchus mykiss*: comparative physiology and molecular evolution. Journal of experimental biology 208, 1951–1961 (2005). 15879075 10.1242/jeb.01551

[b51] Wang L. . Approach on the reference values of serum transaminase in several kinds of cultured fishes. Feed Industry 32, 18–20 (2011).

[b52] Kültz D., Bastrop R., Jürss K. & Siebers D. Mitochondria-rich (MR) cells and the activities of the Na^+^ K^+^-ATPase and carbonic anhydrase in the gill and opercular epithelium of *Oreochromismossambicus* adapted to various salinities. Comparative Biochemistry and Physiology Part B: Comparative Biochemistry 102, 293–301 (1992).

[b53] Xing Y. & Lin J. Application of electrochemical treatment for the effluent from marine recirculating aquaculture systems. Procedia Environmental Sciences 10, 2329–2335 (2011).

