# Supplementary material for: Synergistic Effects of Micro-electrolysis-Photocatalysis on Water Treatment and Fish Performance in Saline Recirculating Aquaculture System
Source: Sci Rep. 2017 Mar 27;7:45066. doi: 10.1038/srep45066 (PMC5366865; doi:10.1038/srep45066)
Supplement: Supplementary Information [file srep45066-s1.doc]

Synergistic Effects of Micro-electrolysis-Photocatalysis on Water Treatment and Fish Performance in Saline Recirculating Aquaculture System

Zhangying Ye^1^, Shuo Wang^1^, Weishan Gao^1^, Haijun Li^1,2^, Luowei Pei^1^, Mingwei Shen^1^, Songming Zhu^1^*

*1. College of Biosystems Engineering and Food Science, Zhejiang University, Hangzhou 310058, China;*

*2.College of Engineering, Anhui Agricultural University, Hefei 230036, China*

*** Corresponding author (e-mail: [zhusm@zju.edu.cn](mailto:zhusm@zju.edu.cn); phone: +86-571-88982373)

**Supplementary Information**

In this part, we mainly researched removal effect of TAN by micro current electrolysis for aquaculture seawater, under different circulating water flow rate, temperature and current density. The preliminary small-scale electrochemical experiment system was as follow^1^.


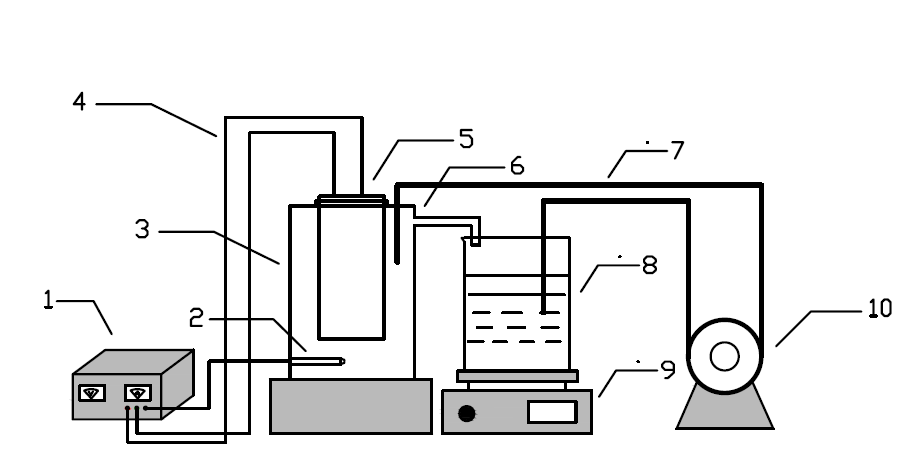


**Fig. A**. Sketch of micro current electrolysis

1. DC power supply 2. reference electrode 3. electrolysis cell 4. supply lead 5. electrode 6. draft tube 7. silicone tube 8. beaker 9. constant temperature blender with magnetic force 10. peristaltic pump

The parameter of electrolysis units was: electrolysis cell (plexiglass, 12.0cm×11.0cm×13.0cm) and two electrode pieces(9.6cm×6.0cm of each). The initial TAN concentration of simulated seawater was 10mg/L and the salinity was 30‰. 2L initial solution of simulated seawater was heated by the constant temperature blender with magnetic force. Until the temperature of the solution reached the set value, pour that into the electrolysis cell and 1L beaker respectively. Then put the electrochemical experiment system into an artificial climate incubator which set the same temperature as the solution. Turn on the peristaltic pump and make the solution circulating in the electrolysis cell and beaker. When the flow rate was stable, it is regarded as the system reaching an equilibrium. Afterwards, regulate current of the power supply, until the current density reached the set value, begin the electrolysis experiment. And sample 5ml solution every 15 minutes from the beaker. Under the different set value of velocity(100mL/min, 200mL/min, 300mL/min),temperature(18℃, 25℃, 32℃) and current density (20A/m^2^, 40A/m^2^, 60A/m^2^), electrolysis experiment time was 90min for the simulated seawater. Determine the corresponding parameters at the end of the experiment. The experimental results are as follows:

a) After electrolysis for the 10mg/L of ammonia concentration solution, the TAN removal rate reached more than 90%. And the final TAN concentration was below 1mg/L, that is, under the harmful concentration for most breeding object.

b) The effect of circulating water velocity and temperature on the removal of TAN was not obvious, and the current density had a significant effect on the removal rate of TAN.

c) The results of orthogonal experiment showed that the main factor for removal efficiency of TAN was the current density. And the interactions between current and temperature and velocity were not significant. Finally, the optimal electrolysis conditions determined for the removal of TAN were current density 40A/m^2^,temperature18℃ and velocity300mL/min.

1. Ye, Z. et al. Ammonia removal effect by using micro-current electrolysis in aquaculture saline water. *Transactions of the Chinese Society of Agricultural Engineering* **32,** 212-217(2016).
